# Sex differences in fatigability after ischemic preconditioning of non-exercising limbs

**DOI:** 10.1186/s13293-020-00338-z

**Published:** 2020-10-27

**Authors:** Hugo M. Pereira, Felipe F. de Lima, Bruno M. Silva, André F. Kohn

**Affiliations:** 1 Department of Health and Exercise Science, University of Oklahoma, 1401 Asp Ave, Norman, OK 73019 USA; 2 Biomedical Engineering Laboratory/EPUSP, University of São Paulo, São Paulo, SP Brazil; 3 Department of Physiology, Federal University of São Paulo, São Paulo, SP Brazil

**Keywords:** Gender differences, Ischemic conditioning, Pain, Plantar flexor muscles, Time to task failure

## Abstract

**Background:**

Ischemic preconditioning (IPC) is suggested to decrease fatigability in some individuals but not others. Sex differences in response to IPC may account for this variability and few studies systematically investigated the effects of IPC in men and women. The goal of this study was to determine if time to task failure, perception of pain, and neuromuscular mechanisms of fatigability were altered by IPC in men and women.

**Methods:**

Ten women (29 ± 5 years old) and 10 men (28 ± 6 years old) performed isometric contractions with the plantar flexor muscles of the dominant leg at 20% of maximal voluntary contraction until task failure. We used a repeated measures design where each individual performed 3 randomized and counterbalanced test sessions: (A) IPC session, cuff inflation and deflation (5 min each repeated 3 times) performed before the exercise by inflating cuffs to the non-dominant leg and arm; (B) sham session, cuffs were inflated for a short period (1 min); and (C) control session, no cuffs were involved.

**Results:**

Compared with control, IPC increased time to task failure in men (mean difference, 5 min; confidence interval (CI) of mean difference, 2.2; 7.8 min; *P* = 0.01) but not women (mean difference, − 0.6 min; CI of mean difference, − 3.5; 2.4 min; *P* = 0.51). In men, but not women, the IPC-induced increase in time to task failure was associated with lower response to pressure pain (*r* = − 0.79). IPC further exposed sex differences in arterial pressure during fatiguing contractions (session × sex: *P* < 0.05). Voluntary activation, estimated with the twitch interpolation technique, and presynaptic inhibition of leg Ia afferents were not altered after IPC for men and women. The tested variables were not altered with sham.

**Conclusions:**

The ergogenic effect of IPC on time to task failure was observed only in men and it was associated with reductions in the perception of pain. This pilot data suggest the previously reported inter-individual variability in exercise-induced fatigability after IPC could be a consequence of the sex and individual response to pain.

## Introduction

Ischemic preconditioning (IPC) consists of repeated brief cycles of ischemia and reperfusion (~ 5 min each) [1]. IPC was originally developed to prevent subsequent ischemia-reperfusion injury in cardiac muscles [2] and later applied to skeletal muscles [3]. Two systematic reviews indicate that IPC applied to the limbs before an exercise has the potential to improve time-trial performance of *dynamic* whole body exercises [4, 5], and subsequently attracted the attention of clinicians because of its potential to improve individual motor performance. During *submaximal isometric* fatiguing contractions that are typically used in clinical settings when movement is contraindicated (e.g., when a cast is being used), there is minimal understanding of the effects of IPC particularly in women. For example, IPC was shown to increase the time to task failure of the knee extensor muscles in men and its effects in women were not investigated [6], which prevents the prescription of evidence-based IPC protocols that are specific for women. During submaximal isometric contractions, women typically have longer time to task failure that parallels sex difference in mechanisms of fatigability, such as lower pressure response [7–9], and these differences between men and women in mechanisms of fatigability may account for some of the inconsistent results regarding the ergogenic effects of IPC [4, 5, 10]. In men, neuromuscular responses frequently associated with fatigability, such as decline in voluntary activation and alterations in muscle contractile properties, were not altered by IPC during *a maximal isometric contraction* sustained for 2 min [11] or after repeated cycling sprints [12]. Sex differences in these variables in response to IPC are not known, and neither the effects of IPC on motor neuron sensitivity to synaptic inputs from peripheral afferents, that are known to be modulated during muscle contractions [13–15].

The ergogenic effects of IPC can occur locally (i.e., in the same limb as the IPC is employed) or at a distance (e.g., in the contralateral limb) [4, 10], and the latter will be employed in the current study to prevent potential nerve block of the exercised leg. In both cases, IPC induces pain during the cycles of ischemia and reperfusion and individuals frequently report greater levels of pain during the first cycle of ischemia compared to the last one [16, 17]. The gradual reduction in pain during the cycles of ischemia and reperfusion resembles the so called conditioned pain modulation mechanism [18], that has typically lower magnitude in women than men [19], and previously shown during ischemia [20]. Conditioned pain modulation, which is the inhibition of pain in one part of the body produced by a noxious stimulus in another part of the body, involves the activation of descending inhibitory pathways with diffuse distribution in the spinal cord [21]. Additionally, given the known association of conditioned pain modulation and cardiovascular responses [22], IPC has the potential not only to alter the response to pain but also the pressor response during fatiguing contraction [23]. In men, IPC was shown to reduce mean arterial pressure at rest [23, 24] but to increase it during a handgrip exercise [25]. It is not known whether the regulation of arterial pressure during fatiguing contractions performed after IPC is different between men and women.

In order to shed some light on the issues raised above, this study aimed to (1) determine sex difference in exercise-induced fatigability after IPC and (2) determine if the alterations in time to task failure after IPC are associated with the perception of pain and/or the involvement of neuromuscular mechanisms and/or alterations in arterial pressure. We assessed voluntary activation, presynaptic inhibition of leg Ia afferents and muscle twitch amplitudes to estimate the contribution of central and peripheral mechanisms on the time to task failure during isometric contractions. It was hypothesized that changes in time to task failure after IPC would parallel sex differences in response to pain, muscle activation and regulation of arterial pressure.

## Methods

### Participants

Ten men (26 ± 7 years) and 10 women (29 ± 6 years) participated in the study. This sample size was estimated a priori using previous data detecting IPC-induced alteration in time to task failure [6], allowing an observed power of at least 80% and alpha of 5% in the current study. The individuals were naive to the protocol, healthy, without any neurological, cardiovascular, or orthopedic condition. Subjects were excluded if they reported acute or chronic pain in any part of the body, current use of analgesics, or psychotropic medications. Use of oral contraceptives was recorded and menstrual cycle phase (follicular vs. luteal) was estimated using the first day of menses as previously reported [26]. Written informed consent, approved by the institutional review board, was obtained from all participants prior to participation in the study.

### Experimental protocol

Participants attended an initial familiarization session followed by three randomized experimental sessions (> 3 days apart). During the familiarization session, participants were assessed for physical activity levels [27] (32.7 ± 24.5 MET-hour/week, without sex difference: *P* = 0.37) and leg dominance [28] (all right-footed). The pain catastrophizing scale [29] was used to investigate any potential influence of coping strategies such as magnification (i.e., exaggerated response), rumination (e.g., “I keep thinking this is terrible”) and helplessness (e.g., “I thought it was never going to get better”), when reporting pain levels. During the familiarization session, the protocol of presynaptic inhibition of leg Ia afferents was performed and each individual practiced maximal and submaximal contractions.

**Fig. 1:**
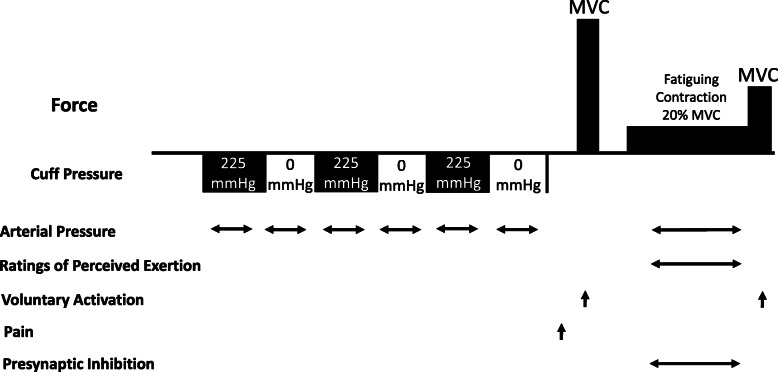
Experimental protocol. Rectangles, above the horizontal line, show the order of isometric force tasks performed with the plantar flexor muscles by each participant. Maximal voluntary contractions (MVC) were performed before and immediately after the fatiguing contraction. Cuff pressure values applied to the non-dominant arm and leg during the ischemic conditioning and sham sessions are shown below the horizontal line. For the *ischemic preconditioning session*, the cuffs were inflated for 5 min and later deflated for 5 min and this was repeated 3 times. During the *sham session*, the cuffs were inflated for only 1 min and later deflated for 9 min and this was repeated 3 times. During the *control session*, each individual sat quietly for the same time period and no cuffs were used. The horizontal arrows indicate variables measured continuously (blood pressure, ratings of perceived exertion, and presynaptic inhibition) and upward arrows indicate times when voluntary activation and pain were assessed. Note that the schematic is not to scale for time or force

After the familiarization session, each participant attended three experimental sessions: control, sham, and IPC. The experimental sessions were counterbalanced among the men and women separately. For each test session, each individual was reminded to avoid caffeine, alcohol ingestion, or consumption of any drug 24 h prior to the test. Each experimental session involved the following procedures (Fig. 1): (1) cycles of ischemia and reperfusion of non-dominant limbs, or quiet rest without the cuffs, depending if the session was control, IPC, or sham; (2) assessment of pain while subjects were at rest with the pain-pressure device (upward arrow in Fig. 1); (3) measurement of maximal voluntary contraction (MVC) of the plantar flexor muscles of the dominant leg. Each MVC trial lasted 3–4 s and individuals received strong verbal encouragement. At least 2 trials of MVCs were performed with 120 s of rest interval between trials. Additional trials were conducted if the participants did not achieve at least 2 MVC torque values within 5% of each other. Participants were provided visual feedback of the ankle torque during each MVC trial; (4) fatiguing contraction at 20% of plantar flexor MVC of the dominant leg. During the fatiguing contraction, each participant matched a horizontal line that was displayed on the monitor and was strongly encouraged to sustain the force for as long as possible. Time to task failure was detected automatically using a custom-written program (LabVIEW; NI, Austin TX, USA) to minimize the influence of transient fluctuations in motor output on task failure. The criterion for time to task failure was when the fatiguing contraction torque fell below 10% of the target force for more than 3 s. All individuals were blinded to the task failure criterion and they were not informed of their time to task failure until completion of their last session. Presynaptic inhibition leg Ia afferents was assessed during the fatiguing contractions; (5) measurement of MVC immediately at task failure.

### Remote ischemic preconditioning and sham

During ischemic preconditioning session, the cycles of ischemia and reperfusion were performed in accordance with previous studies evaluating the effects of the technique on exercise performance [25, 30, 31]. Briefly, after the individuals were positioned in the set up to measure plantar flexion force of the dominant leg, the occlusion cuffs were placed proximally around the non-dominant thigh and upper arm. Cuffs were not used in the dominant limbs to prevent nerve block, which could subsequently alter the tested reflex sensitivity in the exercising limb [32]. The cuff placed around the thigh was customized and had two independent chambers of 36 × 17.5 cm each, arranged in series that covered approximately 80% of the thigh circumference [30]. The arm cuff had a chamber of 27 × 13 cm (Welch Allyn) and also covered approximately 80% of the upper arm. Both cuffs were inflated simultaneously to 225 mmHg for 5 min, and then released for a 5-min recovery period. Three cycles of inflations and deflations were performed before the fatiguing contractions (Fig. 1) [25, 30, 31]. The sham intervention used a strategy previously reported [30]. In brief, the location of the cuffs were the same as described above, but the cuffs were inflated to 225 mmHg for 1 min (not sufficient to change muscle oxygen saturation [33] or provide protection against subsequent ischemia [34]) and deflated for 9 min, so total time was equivalent to those of the ischemic preconditioning session. Shortening cuff inflation time minimized the potential for descending inhibitory systems of pain but allowed adequate placebo effects. Placebo induction was performed by telling the subject that both IPC and sham interventions would improve performance similarly as compared to control. Nocebo avoidance was accomplished by saying that both ischemic preconditioning and sham interventions would cause no harm, despite signs and symptoms of the occlusion compared to control.

### Torque

Subjects were seated comfortably on a customized chair, with armrest and headrest, designed for measuring ankle torque during isolated plantar flexion and dorsiflexion contractions. The setup was previously described [13]. In brief, the subject’s dominant foot was positioned at 90° and firmly strapped, without causing discomfort, to a rigid pedal connected to a force transducer (Transtec N320, Brazil). The transducer output was amplified by a MEB-2300K system (Nihon-Kohden, Japan) and digitized by two systems: (1) a Power 1401 (Cambridge Electronic Design Limited -CED, UK) for offline data processing and (2) a USB-6343 (National Instruments—NI, USA) for online data processing and visual feedback. The sampling frequency in both systems was 5 kHz. A 15-in. monitor placed 1.5 m in front of each participant was used to display the torque. Visual feedback was provided with a constant *y*-axis in a 10-s window. During the contractions, the force feedback consisted of a line moving from left to right.

### Electromyography (EMG)

EMG was used to quantify the reflex responses. The skin was prepared with abrasive gel and the EMG signals were recorded from the dominant leg using bipolar round-shaped surface electrodes (Ag-AgCl, 0.8-cm diameter, with an inter-electrode distance of 2 cm). For the soleus muscle, the electrode with most proximal contact was positioned 3–4 cm beneath the inferior margin of the two heads of the gastrocnemii muscles identified by visual inspection, as commonly used to better visualize H-reflex responses [13, 35]. Bipolar electrodes were also positioned on the tibialis anterior muscle and a reference electrode (round with 3-cm diameter) was placed over the tibia. The EMG signals were amplified and filtered (5 Hz to 2 kHz) by a MEB-2300K system (Nihon-Kohden, Japan) and digitized by two systems: (1) a Power 1401 (Cambridge Electronic Design Limited—CED, UK) for offline data processing and (2) a USB-6343 (National Instruments—NI, USA) for online data processing. The sampling frequency in both systems was 5 kHz.

### Electrical stimulation

Two constant-current electric stimulators (STMISOLA, Biopac, USA) controlled by an USB-6343 interface (NI, USA) and custom-written program in Labview (NI, USA) were used to deliver the stimuli via low impedance carbon adhesive surface electrodes (area = 2cm^2^ for each electrode). One stimulator was used to stimulate the fibular nerve at the fibular head and the other was used to stimulate the tibial nerve at the popliteal fossa [13, 35]. Stimulation locations were determined both at rest and during contractions to take into account changes in muscle geometry during muscle contraction. Electrical stimulation parameters were determined during the familiarization session and confirmed during each experimental session before performing the fatiguing contraction.

#### M-waves

A single pulse (1 ms duration) was used either on the tibial or fibular nerves to elicit the respective M-waves of the plantar flexor and dorsiflexor muscles. The intensity of the stimulation for each nerve was determined by increasing the current until a maximal M-wave amplitude was obtained (Mmax). The stimulation intensity was then increased further by 20% to ensure maximal excitation of the nerve tested. The Mmax amplitudes of the dorsiflexor and plantar flexor muscles were constant across sessions. Test M-waves of 20% of Mmax from both soleus and tibialis anterior muscles were also elicited regularly to secure efficacies of the conditioning and test stimuli.

#### H-reflexes and presynaptic inhibition

H-reflexes were evoked during plantar flexion contractions at 20% of MVC, which minimized the influence of homosynaptic postactivation depression [36, 37]. Control and conditioned H-reflexes, to estimate presynaptic inhibition, were elicited with an interval of 1.75 ± 0.25 s in random order during the contractions. More specifically, the following were performed:

- *Control H-reflexes.* Soleus H-reflexes were obtained in the ascending phase of the recruitment curve with an electrical rectangular pulse (1-ms duration) delivered to the tibial nerve. The stimulation was adjusted to evoke H-reflexes between 20 and 40% of *Mmax*. The amplitude of the control H-reflex was maintained constant within and across sessions by the custom-written LabVIEW program as the sensitivity of the H-reflex to excitatory and inhibitory inputs depend on its amplitude [35]. All evoked potentials were monitored online and stored in a computer for subsequent analysis.
- *Presynaptic inhibition.* The soleus H-reflexes were conditioned by a stimulus applied to the fibular nerve to activate primary afferent depolarization interneurons responsible for presynaptic inhibition of the Ia afferents [38, 39]. The conditioning stimulus was set at 1.1 × motor threshold of the tibialis anterior muscle. The interval between the conditioning stimulation and the soleus stimulation that produced the greatest depression in soleus H-reflex was determined for each participant from 5 to 30 ms (i.e., D1 Inhibition) in 5-ms increments in the familiarization session. There were equal numbers of conditioned and control H-reflexes, so presynaptic inhibition was elicited in 50% of the evoked H-reflexes in random order during the 20% MVC contraction.

#### Voluntary activation

A supramaximal doublet stimulus (10-ms interpulse interval) was delivered to the tibial nerve during the MVC plateau for the purpose of eliciting a superimposed twitch and also ~ 1 s following each MVC with the muscle at rest (resting twitch) (Fig. 1). Participants were well familiarized with these procedures and were provided visual feedback and strong verbal encouragement during the MVCs. Voluntary activation measurement was shown to be reproducible in this muscle group [40].

### Perception of effort

A modified Borg 10-point scale was used to assess perception of effort of the lower leg during the fatiguing contractions. The scale was anchored so that “1” was complete rest and “10” corresponded to the hardest effort to perform a muscle contraction with the plantar flexor muscles. Rating of perceived exertion (RPE) was recorded every minute during fatiguing contraction until task failure.

### Pain

A visual analog scale (VAS) was used to access pain. The scale was anchored so that “0” represented no pain and “10” corresponded to the worst pain [41]. This scale was used to access pressure-pain perception during a test performed with a custom-made device similar to one used in a previous report [42]. In brief, a 1-kg mass was placed on the dorsum of the middle finger of the dominant hand for 2 min. The device consisted of a small block with an edge of 8 × 1.5 mm that was 3D printed in polylactide, and the mass was positioned between the proximal and distal interphalangeal joints. During the pressure-pain test, each individual was asked to rate the intensity of pain every 20 s using the VAS described above. Participants were informed they could discontinue the test at any time if they reached pain tolerance. The pain test was applied after the cycles of ischemia and reperfusion and before the MVC assessment in every session (represented by an upward arrow in Fig. 1 and detailed in experimental protocol).

### Arterial pressure

Arterial pressure was monitored with an automated wrist cuff (BP652N; OMRON Electronic Components, Lake Forest, IL, USA). The cuff was placed around the wrist of the dominant arm, with the hand placed on a table adjacent to the subject at heart level. Arterial pressure and heart rate (HR) were monitored and documented every minute during the cycles of ischemia and reperfusion (or rest during control session), and during fatiguing contractions at the start of the contraction and every minute thereafter until task failure.

### Data analysis

All H-reflex responses were extracted from the interference EMG and characterized by the peak-to-peak amplitude. Five epochs (i.e., the start of the task, 25%, 50%, 75% and 100% of time to task failure) were used to quantify H-reflex amplitudes during the fatiguing contractions. For each epoch the amplitude of the control H-reflex and conditioned H-reflexes (D1 Inhibition) were obtained by averaging 10 responses of each reflex. The magnitude of presynaptic inhibition was expressed in percentage [% presynaptic inhibition = (1 − (conditioned H-reflex amplitude/control H-reflex amplitude)) × 100], so that greater values indicate greater inhibition. Technical difficulties prevented obtaining H-reflex during fatiguing contractions in one man, however data from other variables (i.e., time to task failure, pain, arterial pressure, voluntary activation) from this participant were included in the analysis. The level of voluntary activation was quantified using the interpolated twitch technique. Any evoked increase in torque of the plantar flexor muscles during a contraction (superimposed twitch) was expressed as a percentage of the torque from the same stimulus evoked in the potentiated relaxed muscle (resting twitch) [% activation = 1 − (superimposed twitch/resting twitch) × 100] [40]. Mean arterial pressure (MAP) was calculated with the following equation: MAP = Diastolic arterial pressure + [1/3 + (Heart rate × 0.0012)] × (Pulse pressure) [43].

### Statistical analysis

Data are shown as mean and SD in the text. The dependent variables time to task failure, MVC, resting twitch amplitude, pain assessed with the pain-pressure device, mean arterial pressure, and rating of perceived exertion were analyzed with separate repeated measures analysis of variance (ANOVA) with sex as a between-subject factor. Repeated measures included sessions (control, sham, IPC), time either during either fatiguing contraction (0, 25, 50, 75, and 100% of time to task failure) or pain-pressure test (20, 40, 60, 80, 100, 120 s). The statistical design was session × sex for time to task failure and session × time × sex for the remaining variables listed above. To meet normality assumption data was transformed if necessary. For example, presynaptic inhibition data was logarithm transformed and voluntary activation, which is often left skewed, was inverse sine transformed. Data was converted back to original scale for interpretation. For each ANOVA the sphericity of data was verified with Mauchly’s test and the Greenhouse-Geisser correction was applied whenever necessary. Variables from the pain catastrophizing scale were compared between men and women with Mann-Whitney *U* test. Because the change in pain ratings after IPC was not normally distributed for women according to the Shapiro-Wilk test, the Spearman’s rank correlation coefficient was used to determine its association with changes in time to task failure after IPC. Level of significance was *P* < 0.05, and the analyses were performed in IBM statistical package for social sciences (SPSS) version 24. Further interpretation of the influence of IPC on time to task failure was performed using the Hedge’s *g* effect size and its confidence interval, as it is adequate for the sample size used in the current study [44].

## Results

### Time to task failure

**Fig. 2:**
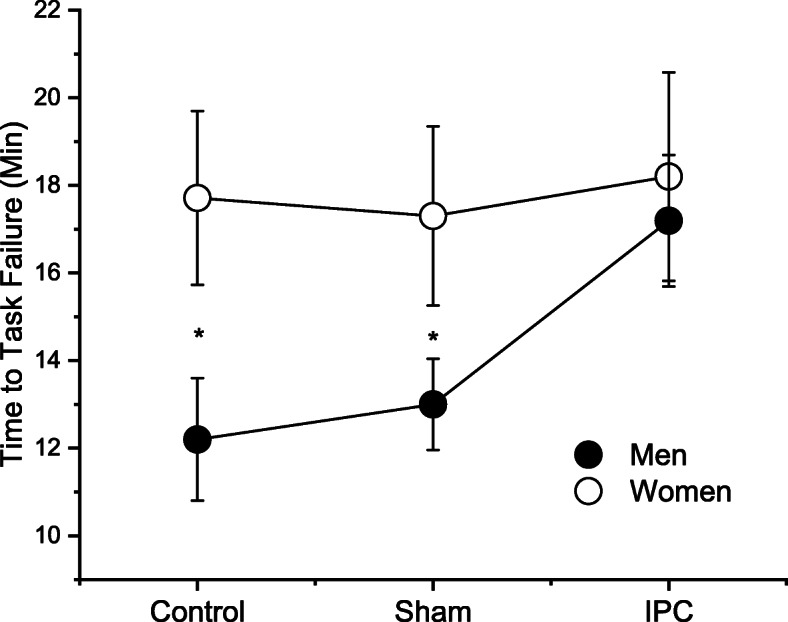
Average of time to task failure during the control, sham, and ischemic preconditioning (IPC) sessions for men and women. IPC increased the time to task failure for men, but not women (session × sex contrast: *P* = 0.02). Asterisk indicates sex difference. Data is from 10 women and 10 men

During the control session women had greater time to task failure than men (sex effect: *P* = 0.03) (Fig. 2). The time to task failure was altered by IPC with different responses in men and women (session × sex: *P* = 0.03). More specifically, compared to control, the time to task failure was increased following IPC in men (12.2 ± 4.9 vs. 17.2 ± 4.8 min respectively, *P* = 0.01; effect size, 0.96 [confidence interval (CI) of effect size, 0.03; 1.88]), but not women (17.7 ± 6.2 vs. 18.3 ± 7.5 min, respectively, *P* = 0.51; effect size, 0.08 [CI of effect size, − 0.79; 0.96]) (Fig. 2). The effect of sham on time to task failure was trivial and without statistical significance for men (13.1 ± 3.3 min; effect size, 0.20 [CI, − 0.68; 1.08]) or women (17.3 ± 6.5 min; effect size, − 0.06 [CI, − 0.94; 0.82]) compared to control (both sexes with *P* > 0.05). Adding the menstrual cycle phase or presence of oral contraceptives into the statistical model did not alter these results (main effect of menstrual cycle or oral contraceptives and interactions, all with *P* > 0.05).

### Maximal voluntary contraction

#### Before fatiguing contraction

There was no statistical difference in MVC across sessions for men and women (both session and session × sex: *P* > 0.05) and men were stronger than women (129.9 ± 19.4 vs. 91.7 ± 19.4 Nm respectively; sex effect: *P* < 0.01).

#### At task failure

MVC was lower compared with baseline (time effect: *P* < 0.01) for both men and women (73.3 ± 26.8 vs. 54.3 ± 14.4 Nm, respectively, time × sex: *P* = 0.13). There was no statistical differences in MVC at task end across sessions (session effect: *P* = 0.73) for men and women (session × sex: *P* = 0.41).

### Voluntary activation

#### Before fatiguing contraction

Voluntary activation was similar between men and women (95.8 ± 6 vs. 92.7 ± 6%, respectively; sex effect: *P* = 0.19) and the values were not different across sessions, and without interaction (both session effect and sex × session: *P* > 0.05).

#### At task failure

Fatiguing contraction reduced the voluntary activation (fatigue effect: *P* < 0.01) for control, sham and IPC for men (77.3 ± 16.1 vs. 76.9 ± 16.3 vs. 82.6 ± 14.8%, respectively) and women (79.3 ± 16.1 vs. 83.9 ± 16.3 vs. 79.4 ± 14.8%, respectively) (session effect: *P* = 0.44; session × sex: *P* = 0.34).

### Resting twitch amplitude

#### Before fatiguing contraction

The amplitude of the potentiated twitch torque was greater for men than women (17.2 ± 4 vs. 13.6 ± 4 Nm, respectively; sex effect: *P* = 0.04). There was no difference in evoked twitch amplitude across sessions (session effect: *P* = 0.19) for men and women (session × sex: *P* = 0.79).

#### At task failure

The potentiated twitch amplitude was lower at task end compared with baseline (time effect: *P* < 0.01) without differences for men and women (13.9 ± 3.6 vs. 9.9 ± 3.6 Nm, respectively; time × sex: *P* = 0.72). On average, there was no difference across sessions in the reductions of twitch amplitude (session × time: *P* = 0.78) for men and women (session × time× sex: *P* = 0.79).

### Presynaptic inhibition (D1 inhibition) and control H-reflex

IPC or sham had no effect on the magnitude of presynaptic inhibition (session effect: *P* = 0.23) and there was no interaction (session × sex: *P* = 0.72; session × sex × time: *P* = 0.81). During the control session, there was no sex difference in magnitude of presynaptic inhibition during the fatiguing contraction (average of all data points during the fatiguing contraction, 34.6 ± 24 vs. 33.5 ± 16 % of control H-reflex, respectively, sex effect and time × sex: *P* > 0.05).

There was no change over time of the control H-reflex (i.e., no hysteresis) (time effect: *P* = 0.59) for men or women (time × sex: *P* = 0.29). For the control H-reflex there was no statistical difference between control, sham and IPC sessions (average of all data points during the fatiguing contraction, 30.5 ± 3.0 vs. 30.6 ± 2.2 vs. 29.2 ± 2.61 % of *Mmax,* respectively; session effect: *P* = 0.24) for men and women (sex effect: *P* = 0.46) and no interaction (session × sex, session × time, session × time × sex: all with *P* > 0.05).

### Pain

#### Pain catastrophizing

The total score on the pain catastrophizing scale was not statistically different between men and women (16.6 ± 5 vs. 13.8 ± 6 au, respectively, *P* = 0.39). There was no difference between men and women in the subscales of magnification (4.4 ± 2 vs. 3.1 ± 2 au, respectively, *P* = 0.22), rumination (6.1 ± 2 vs. 5.6 ± 2.5 au, respectively, *P* = 0.63), and helplessness (6.1 ± 2 vs. 5.1 ± 2.5 au, respectively, *P* = 0.35).

#### Pain ratings at rest assessed by the pain-pressure device

**Fig. 3:**
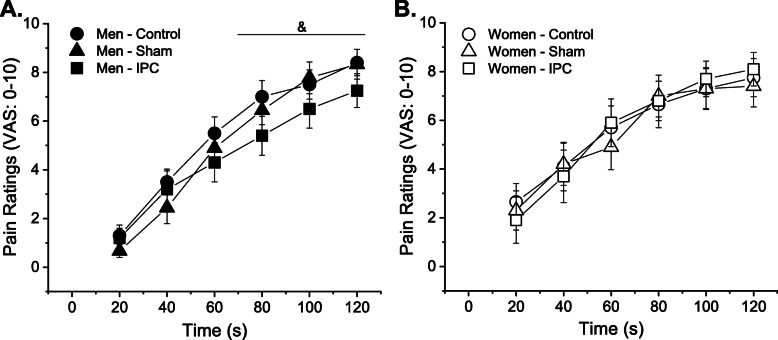
Pain ratings assessed with the pain-pressure device in men (**a**) and women (**b**). Over time men had lower pain ratings during IPC session compared with control whereas women did not show changes in pain ratings across sessions (session × time × sex: *P* = 0.01). Sham session did not alter pain ratings in men or women. VAS, visual analog scale; IPC, ischemic conditioning technique. Ampersand indicates session × time × sex: *P* < 0.05. Data is from 10 women and 10 men

Men and women had distinct pain ratings over time across sessions (session × time × sex: *P* = 0.02) (Fig. 3). More specifically, post hoc comparisons indicate the pain ratings were lower after IPC compared with control in men but not women (session × time × sex: *P* = 0.01). Post hoc analyses also indicate that sham did not alter pain ratings compared with control in men or women (session × time × sex: *P* = 0.31). During the control session, there was no sex difference in pain ratings (sex effect: *P* = 0.99, time × sex: *P* = 0.18).

### Ratings of perceived exertion (RPE)

RPE increased during fatiguing contraction (time effect *P* < 0.01) for men and women (time × sex: *P* = 0.91). There was no statistical difference in RPE across sessions (session effect: *P* = 0.08) for men and women (session × sex: *P* = 0.77; session × sex × time: *P* = 0.26).

### Arterial pressure

#### At rest

**Fig. 4:**
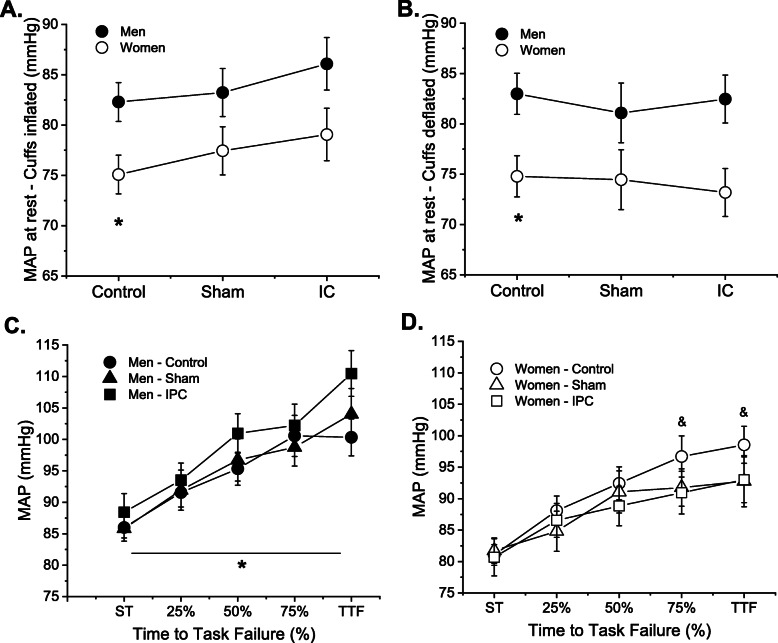
Mean arterial pressure (MAP) at rest when cuffs were inflated (**a**) and deflated (**b**) and during fatiguing contractions (**c**, **d**) for each test session. Men had greater MAP than women during control session at rest or during fatiguing contraction (sex effect: *P* < 0.05). MAP increased for both men and women when cuffs were inflated in sham or IPC session compared with control session (**a**). When cuffs were deflated, IPC and sham sessions did not alter MAP compared with control for men or women (**b**). During fatiguing contractions MAP was greater during IPC session compared with control for men (**c**), whereas women had lower MAP at IPC compared with control (**d**). Asterisk indicates sex difference during control session (*P* < 0.05). Ampersand indicates sex difference of the IPC session compared with control session (i.e., session × sex effect: *P* < 0.05). IPC, ischemic conditioning technique. Data is from 10 women and 10 men

MAP was greater when cuffs where inflated compared with deflated (main effect of ischemia: *P* < 0.001) for both men and women (ischemia × sex: *P* = 0.12). MAP had a different response across sessions for men and women when cuffs were inflated and deflated (session × cuff condition × sex: *P* = 0.02).The description of each condition is as follow:

- Cuffs inflated: Men had greater MAP than women during the control session (82.3 ± 7 vs. 75.1 ± 5 mmHg; *P* = 0.02). Post hoc analyses indicate that IPC increased MAP compared with control (mean difference, 4 mmHg; *P* < 0.01), but not sham (mean difference, 1.6 mmHg) with similar trends in men and women (session × sex: *P* = 0.82) (Fig. 4a).
- Cuffs deflated: Men had greater MAP than women during the control session (82.9 ± 8 vs. 74.8 ± 5 mmHg; *P* = 0.01). IPC or sham did not alter MAP compared with control (session effect: *P* = 0.59), for men or women (session × sex: *P* = 0.65) (Fig. 4b).

#### During fatiguing contractions

Men had greater MAP than women (average of all data points, 96.4 ± 7 vs. 89.3 ± 7 mmHg, main effect of sex: *P* = 0.03). MAP increased during fatiguing contraction (time effect: *P* < 0.01) with different trends between men and women across sessions (session × sex: *P* = 0.02; session × sex × time: *P* = 0.04). Compared with control, the MAP during IPC was greater for men but lower for women particularly at 75% and 100% of time to task failure (session × sex: *P* = 0.01). Sham session had no effects on MAP compared with control in men and women (both session effect and session × sex: *P* > 0.05) (Fig. 4c, d).

### Associations

**Fig. 5:**
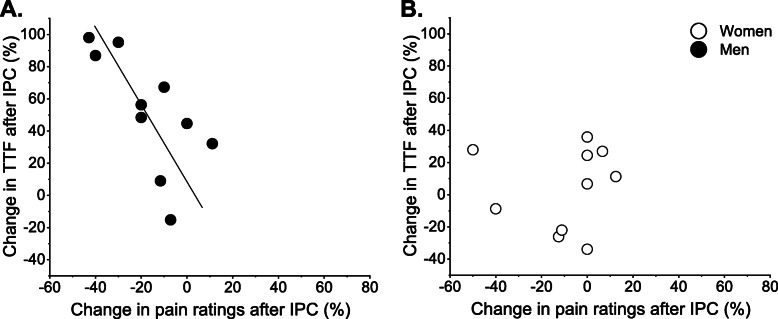
Association between the change in time to task failure after IPC and change in pain ratings. The percentage increase in time to task failure after IPC was associated with reduction in pain ratings after IPC for men (**a**) but not women (**b**) (positive values on *y*-axis represent increase in time to task failure after IPC and negative values on *x*-axis represent decline in pain ratings after IPC). TTF, time to task failure; IPC, ischemic preconditioning technique

The relative increase in time to task failure after IPC [(increase in time to task failure with IPC/time to task failure of the control session) × 100] was associated with a percentage decrease in level of pain perception assessed with the pain-pressure device [(pain assessed after the IPC technique/pain assessed during control session) − 1 × 100] for men (*r* = − 0.79; *P* < 0.01), but not women (*r* = 0.18; *P* = 0.63) (Fig. 5a, b). There were no associations between changes in time to task failure with changes in presynaptic inhibition, voluntary activation or arterial pressure (all with *P* > 0.05).

## Discussion

The findings of this study are novel and indicate that IPC applied in the non-exercising leg and arm increased the time to task failure of plantar flexor muscles in men but not women (Fig. 2). IPC induced a reduction in response to pressure pain that was associated with the greater time to task failure in men but not women (Fig. 5a, b). Greater time to task failure in men via pain related mechanism is a potential alternative to previous hypothesis suggesting that benefits of IPC could involve improvements in endothelium function or energy metabolism [1, 3]. Placebo induction during sham session had no statistically significant effect in time to task failure, pressure pain after IPC, voluntary activation, or presynaptic inhibition in both men and women. Arterial pressure responses were not altered by IPC when men or women were at rest; however, IPC further exposed sex differences in pressure responses during the fatiguing contraction (Fig. 4c, d). It is also noteworthy that women had greater time to task failure than men during the control session, which is in accordance with previous reports in other muscle groups during isometric contractions, but with limited evidence in the plantar flexor muscles (for review see Hunter [45]).

### Mechanisms of parallel changes in pain ratings and time to task failure

During control session (i.e., without IPC related noxious stimuli), there was no sex difference in pain ratings assessed with the pressure-pain device; however, pain ratings were reduced only in men after the IPC protocol (Fig. 3a, b). These results are consistent with sex differences in conditioning pain modulation and suggest activation of descending inhibitory systems reducing the activity of spinal nociceptive neurons in men but not women [18, 19]. Men who had greater reduction in response to pressure pain during IPC session compared with control also increased the time to task failure after IPC (Fig. 5), and this association was not observed in women. Sham session did not evoke conditioned pain modulation in men or women. Although not directly measured in the current study, the concentration of monoamines and endogenous opioids that are frequently associated with pain modulation [21] was potentially altered during the IPC session with repercussions for exercise performance. Accordingly, naloxone, an opioid antagonist, was shown to impair exercise performance by reducing the maximal power and shortening the time to failure during an incremental cycling test (sex differences were not investigated) [46]. Additionally, fentanyl, an opioid agonist, was shown to increase cycling power in men [47].

In the current study, although conditioned pain modulation response was elicited in men after IPC, D1 presynaptic inhibition during the fatiguing contractions was not altered across sessions for men and women. Others have observed an increase in presynaptic inhibition of group Ia afferents right after application of a chemical nociceptive stimulation when men and women were at rest (sex differences not tested), but the effect of conditioned pain modulation on presynaptic inhibition was not investigated [48]. In animals, the descending modulation of nociceptive pathways was shown to increase presynaptic inhibition of sensory inputs and reduce mechanical pain [49]. The putative alterations in presynaptic inhibition after conditioned pain modulation are yet to be fully understood in men and women.

### Group response to IPC and task specificity

Modulation of central mechanisms associated with fatigability such as voluntary activation and presynaptic inhibition [14, 50] were not altered by IPC in the current study. This is in accordance with others who showed no effects of a single session of IPC on voluntary activation or H-reflex amplitude of the quadriceps muscles in men, but its effects in women were not evaluated [11, 12, 51, 52]. Nonetheless, it is suggested that some individuals seem to respond to the IPC technique but others do not [10, 52, 53] and differences in the populations involved, the intensity of contraction and muscles recruited are some factors that may explain the contradictory findings across studies. For example, physical activity levels were suggested to negatively influence the response to IPC [10]. Stroke survivors, a population that is typically less physically active than healthy individuals, had a reduction in motor unit recruitment threshold during submaximal contractions after IPC [54], which supports the modulation of the central nervous system after IPC. There was no sex difference in physical activity levels in the current study, and therefore this variable likely does not explain the sex difference in response to IPC in our sample. The current study agrees with others that investigated the effects of IPC during fatiguing tasks at submaximal intensities using different muscle groups [6, 52], but it is in contrast with others employing a maximal intensity task (e.g., all out sprint) in men [11, 12, 51] and women [55], and suggests the effects of IPC may be task dependent.

### Cardiovascular response and time to failure after IPC

Sex differences in cardiovascular response after IPC is not completely understood and previous observations of IPC effects on the control of arterial pressure in men were equivocal. More specifically, during a handgrip task some observed lower arterial pressure after IPC [23], whereas others reported no change in arterial pressure or muscle sympathetic nerve activity [56]. In the current study, the increase in time to task failure after IPC in men, but not women, paralleled sex differences in MAP during the fatiguing task. More specifically, men had greater MAP during the fatiguing contraction in the IPC session compared with control or sham sessions, whereas women showed lower MAP values at 75 and 100% of time to task failure during IPC compared with control (Fig. 4c, d). Sex difference in MAP in the IPC session of the current study resembles the greater cardiovascular work during other painful stimuli used to assess conditioned pain modulation [22] and are consistent with previous reports showing increased activation of the sympathetic nervous system in men but not women during painful stimulation [57, 58].

Although greater MAP during the fatiguing contraction may indicate greater input from group III/IV muscle afferents, which is in turn suggested to inhibit central drive to ipsilateral muscles [59, 60], in the current study, men had greater MAP during the fatiguing contraction in the IPC session compared with control and also had greater time to task failure after IPC. Potentially, the IPC-induced changes in the pain perception and concentration of endogenous opioids disrupted the group III/IV muscle afferent feedback loop. Accordingly, we did not observe differences in voluntary activation at task failure across sessions, indicating that the central drive at task failure during IPC session was similar to control despite the greater MAP during IPC.

### Perspectives and significance

Understanding the effects of IPC on sex differences in exercise-induced fatigability is essential when designing training and rehabilitation programs that are specific for men and women. Data from the current pilot study suggest that inter-individual variability in exercise performance after ischemic preconditioning shown in previous reports [4, 5, 61] could be a consequence of the sex and individual response to pain.

The present study investigated the effects of IPC on a limited sample of healthy young adults. Further studies should extend the findings reported here to individuals with chronic diseases and explore any putative implications for rehabilitation of these individuals. Further studies should also investigate the association between reduction in pain after IPC and increased time to task failure in men and its mechanisms, as well as assessing the additive effect of multiple sessions of IPC employed by others during non-fatiguing exercises [62–65].

## Conclusion

IPC applied in the contralateral arm and leg induced alterations in the time to task failure, response to pain, and MAP, with different responses in men and women. Reductions in pain after IPC were associated with greater time to task failure in men but not women. Future studies assessing the effects of IPC on exercise-induced fatigability should consider testing for sex differences and assessing pain related mechanisms.

## Data Availability

Dataset supporting the conclusions of this article is available at 10.6084/m9.figshare.12407795.v1. Additional information is available upon reasonable requested.
